# Comment on “Accentuation of Tumor Growth Secondary to Morphine Administration: The Proneoplastic Role of Morphine besides Its Role in Pain Management”

**DOI:** 10.1155/2012/963492

**Published:** 2012-05-23

**Authors:** Shailendra Kapoor

**Affiliations:** 2300 E Cary Street, Richmond, VA 23111, USA

I read with great interest the recent article by Luk et al. in a recent issue of your esteemed journal [1]. The article is highly thought provoking. Interestingly, the past few years have clearly revealed the connection between morphine administration and tumor development and progression.

For instance, morphine acts on the *μ*-opioid receptors and increases intracellular cyclin D1 and activates G protein receptors resulting in accentuation of the mitogen-activated protein kinase (MAPK) pathway and thereby enhances angiogenesis in breast tumor cells [2]. In fact Farooqui et al. have recently demonstrated that morphine administration in breast tumor animal models activates prostaglandin E_2_ thereby enhancing tumor metastasis also. Similar effects are noted in prostate cancer tissue [3]. For instance, patients who undergo radical prostatectomy for prostate carcinoma and who receive opioid treatment in conjunction with general anesthesia have a 57% higher risk of tumor recurrence in comparison to patients who receive general anesthesia in conjunction with epidural anesthesia [4]. Similarly, Mathew et al. have shown that morphine administration enhances tumor growth in lung cancer models [5]. 

Interestingly, methylnaltrexone inhibits activation of RhoA by inhibiting transactivation of VEGF receptors [6]. As a result it attenuates morphine-induced angiogenesis and thus inhibits tumor growth. PD98059 is another new agent that inhibits the MAPK pathway and thereby decreases opioid-induced tumor cell proliferation [7]. Similarly, celecoxib attenuates opioid-induced stimulation of cyclooxygenase-2 receptors and thereby exerts antineoplastic effects [3]. 

These examples clearly illustrate the role of morphine in enhancing tumor angiogenesis and growth. Clearly, there is a further need to identify further such opioid receptor antagonists besides methylnaltrexone that can inhibit morphine-mediated carcinogenesis.
